# Estrogen, Estrogen Receptor and Lung Cancer

**DOI:** 10.3390/ijms18081713

**Published:** 2017-08-05

**Authors:** Li-Han Hsu, Nei-Min Chu, Shu-Huei Kao

**Affiliations:** 1 Ph.D. Program in Medical Biotechnology, College of Medical Science and Technology, Taipei Medical University, Taipei 110, Taiwan; lhhsu@kfsyscc.org; 2 Division of Pulmonary and Critical Care Medicine, Sun Yat-Sen Cancer Center, Taipei 112, Taiwan; 3 Department of Medicine, National Yang-Ming University Medical School, Taipei 112, Taiwan; 4 Department of Medical Oncology, Sun Yat-Sen Cancer Center, Taipei 112, Taiwan; nmchu@kfsyscc.org; 5 School of Medical Laboratory Science and Biotechnology, College of Medical Science and Technology, Taipei Medical University, Taipei 110, Taiwan

**Keywords:** epidermal growth factor receptor, estrogen, estrogen receptor, hormone, lung cancer, lung adenocarcinoma

## Abstract

Estrogen has been postulated as a contributor for lung cancer development and progression. We reviewed the current knowledge about the expression and prognostic implications of the estrogen receptors (ER) in lung cancer, the effect and signaling pathway of estrogen on lung cancer, the hormone replacement therapy and lung cancer risk and survival, the mechanistic relationship between the ER and the epidermal growth factor receptor (EGFR), and the relevant clinical trials combining the ER antagonist and the EGFR antagonist, to investigate the role of estrogen in lung cancer. Estrogen and its receptor have the potential to become a prognosticator and a therapeutic target in lung cancer. On the other hand, tobacco smoking aggravates the effect of estrogen and endocrine disruptive chemicals from the environment targeting ER may well contribute to the lung carcinogenesis. They have gradually become important issues in the course of preventive medicine.

## 1. Introduction

Estrogens are steroid hormones. 17-β-Estradiol (E2) is the primary reproductive hormone synthesized in the ovary under the stimulation of the follicular stimulating hormone and the luteinizing hormone [1,2,3,4]. Estrone and estriol are mostly synthesized in the liver from E2. The functions of estrogen and its receptors in reproductive organs, especially in a female, have been known for several decades. The importance of the estrogen signaling pathway in various physiologic, pathologic functions and carcinogenesis has also been extensively investigated, especially in the context of breast cancer.

There are two types of classical estrogen receptor (ER). ER alpha (ERα, also known as ESR1), product of genes on chromosome 6, first cloned in 1986 and distributed in breast, ovary, and endometrium. ER beta (ERβ, also known as ESR2), product of genes on chromosome 14, discovered in 1996 [5], with a wider distribution including the bone, brain, colon, endothelium, kidney, lung, ovary, prostate, and testes. They share similar structures and are composed of five domains. The A/B domain is the site of the transcriptional activation, with the coactivator, AF-1. The C-domain is the DNA-binding site. The D-domain hinge contains a nuclear localization signal. The E-domain is the ligand binding domain and the site of the transcriptional activation, with the coactivator, AF-2. The F-domain may play a complex regulatory role. The 55% homology between the ERα and ERβ in the ligand binding domain results in the variable affinities. While both exhibit similar affinities to E2, ERα has a higher affinity to estrone and ERβ has a higher affinity to estriol. In addition to the wide-type ERs, several splicing variants or isoforms of the ERs have been described with variable DNA- or ligand-binding properties.

Upon binding with estrogen, the ERs form either homo- or heterodimers and bind to the estrogen responsive element, the ERE within the promoter of a target gene, and then regulate its transcription in the case of the genomic pathway. ERs may also regulate gene expression via the binding to other transcription factors such as the activator protein 1, AP-1 or the stimulating protein 1, Sp1. On the other hand, ERs may translocate to the membrane, where they may mediate a non-genomic pathway that results in more rapid responses, such as the activation of protein kinase, the production of second messengers, or the regulation of ion channels.

Lung cancer is a leading cause of cancer-related mortality worldwide, including Taiwan. Studies conducted in Western countries estimated that 85–90% of lung cancer cases were attributed to smoking [6,7]. Although 80% of female lung cancer patients worldwide have smoked, less than 10% of Taiwanese women are smokers [8]. In our previous lung cancer study, only 6.4% of the female patients had smoked cigarettes at some time in their lives [9]. There is a lung adenocarcinoma epidemic with an equal occurrence and prognosis in both genders who have never smoked in Taiwan [10]. Smoking history appeared to be a poor prognostic factor for patients with lung adenocarcinoma, rather than as a risk factor. The low smoking prevalence and high incidence rate of adenocarcinoma constituted distinctive characteristics of lung cancer in Asian countries, and leads to the suggested existence of non-tobacco related risk factors in the pathogenesis of lung adenocarcinoma.

Another study showed a more significant survival advantage for elderly women with lung adenocarcinoma, as compared with their male counterparts [11]. In addition to the inferior survival of elderly male patients attributed to the accumulated adverse effect of a higher prevalent smoking habit, the superior survival of the postmenopausal female patients was possibly due to the less estrogen cancer promoting effect. The premenopausal women, who comprised one-fifth of the non-smoking female patients with lung adenocarcinoma, were found to have had the more advanced disease and a shorter survival rate than the postmenopausal women. The epidemiology results suggest that estrogen adversely affects the prognosis of patients with lung adenocarcinoma.

Estrogen is speculated as playing an important role in lung carcinogenesis [12,13]. In healthy lung tissue, ERβ is highly expressed in pneumocytes and in the bronchial epithelial cells, and is required for the maintenance of the extracellular matrix of the lung [14,15]. The lung tissues of ERβ null mice were found to have a decreased number of alveoli and a lesser amount of surfactant [16]. Studies of ER deficient mice have shown that ERα mediates the determination of the alveolar number and the surface area while ERβ affects the lung tissue elastic recoil [17]. Estrogen receptors (ER) are consistently found in lung cancer tissues and cell lines, especially adenocarcinoma, and mostly in the form of the ERβ [18,19,20,21]. Estrogen has been reported to adversely affect the prognosis of lung cancer patients [22,23,24,25,26,27,28,29,30]. However, there are several studies with conflicting results about the effect of estrogen on the risk and/or survival of lung cancer [31,32,33,34,35,36].

We demonstrated ERβ was the predominant ER in the A549 and PE089 lung cancer cell lines, and malignant pleural effusions from the patients with lung adenocarcinoma. Osteopontin (OPN) is a small integrin-binding ligand *N*-linked glycoprotein regulating signaling pathways involved in tumor progression and metastasis [37,38,39]. Enhanced OPN expression has been noted in the plasma of advanced lung cancer patients, and OPN has also been speculated to be involved in the formation of malignant pleural effusion [40,41]. Estrogen up-regulated the OPN expression and promoted lung cancer cell migration via the ERβ activation of the MEK/ERK signaling pathway. An additive effect of the ER antagonist and the epidermal growth factor receptor (EGFR) antagonist on the inhibition of lung cancer cell migration was also observed. Osteopontin supposedly contributes to the cross-talk between the ER and EGFR signaling pathways [11]. In current clinical practice, breast cancer survivors offer a unique patient cohort to evaluate the effect of anti-estrogen on the survival of lung cancer patients [42,43]. We have evaluated the outcome of 26 women who have had second primary lung cancer among 6361 breast cancer patients diagnosed and treated between January 2000, and December 2009, at Sun Yat-Sen Cancer Center and found that the patients who were treated with anti-estrogens for breast cancer had a longer cancer-specific survival rate than those without anti-estrogens [44]. Multivariate analysis confirmed that the anti-estrogen treatment was an independent prognostic factor. These findings reinforced the evidence that estrogen had, in fact, contributed to the lung cancer progression.

This review aims to summarize the current knowledge with regard to the expression and prognostic implications of the ERs in lung cancer, the effect and signaling pathway of estrogen on lung cancer, the hormone replacement therapy and lung cancer risk and survival, the mechanistic relationship between the ER and the EGFR, and the relevant clinical trials combining the ER antagonist and the EGFR antagonist, to investigate the role of estrogen in lung cancer. Interaction between tobacco smoking and estrogen, and the role of endocrine disruptive chemicals targeting ER from the environment in the lung carcinogenesis were also discussed from the viewpoint of preventive medicine. As small cell lung cancer (SCLC) is a distinct neuroendocrine tumor, composed of about 10% to 15% of lung cancer, and the association between estrogen and SCLC was scarcely studied and mostly obsolete [45,46]. The following issues will focus on the non-small cell lung cancer (NSCLC), being mostly adenocarcinoma.

## 2. Estrogen Receptor in Lung Cancer

Baik et al. have systemically reviewed the detection rates of the ERα and ERβ in lung cancer [47,48]. For the ERα, the detection rate using 1D5 with the epitope in N-terminus is 0% to 55%, in contrast with 36% to 84% using HC-20 with the epitope in C-terminus, and 0% to 78% using 6F11 for the full length. For the ERβ, the detection rate using H-150 or 14C8 with the epitope in N-terminus is 49% to 98%, and 16% to 86%, respectively. The detection rate is 9% to 84% using PPG5/10 with the epitope in the C-terminus. The results were variable. Such an inconsistency may be due to the differences in the methodology, i.e., which antibody is used, heterogeneous definitions of positivity, and various patient populations, i.e., pathology, stage, gender, and smoking history [47,48,49]. The ERα antibody with epitope in the C-terminus reported a higher detection rate than that with epitope in the N-terminus in the NSCLC, and was mostly cytoplasm-located. The ERα probably occurs as the N-terminal deleted mutants in the NSCLC and lacks the nuclear localization [47,48,49]. Unlike the ERα, both of the full-length and splicing variants of the ERβ exist in the NSCLC cells. A strong expression of the ERβ was observed in the cytoplasm as well as the nucleus. Standardized measurement, i.e., which antibody was used, or a different approach from immunohistochemistry, e.g., western blot, mRNA expression by real time quantitative PCR, is necessary to make the ERs as useful biomarkers in the future.

Estrogen receptor β appears to be the predominant form in lung cancer from the literature [18,19,20,21]. Five splicing variants had been identified with ERβ1 being the only full-length receptor able to bind ligand and form homodimers in human. The rest of the isoforms are inactive, but they can form heterodimers with ERβ1 to regulate its transcriptional activity [50].

The expressions of ERα and ERβ as a prognosticator for NSCLC have been reported in several studies [20,21,51,52,53,54,55,56,57,58,59,60,61,62,63,64] (Table 1). Contrary to that in breast cancer, ERα in lung cancer was mainly observed in the cytoplasm and associated with a poor prognosis. Most reports found that the nuclear ERβ was predictive of a better prognosis, and the cytoplasmic ERβ was associated with a poor prognosis [47,48]. Nonetheless, opposing results have also been reported [54,60,62,63,65,66]. Co-expression of the cytoplasmic ERβ and the nuclear ERβ that had been reported correlated with a poor survival rate when compared to those without co-expression [67]. The nuclear and cytoplasmic ERs may have a distinct function and affect the prognosis differentially via the genomic or non-genomic pathway. ERβ has also been shown to localize with the mitochondria in a ligand-dependent or -independent manner and can affect the bioenergetics and anti-apoptotic signaling. Mitochondrial ERβ sequesters Bad and inhibit Bad-Bcl-XL, and Bad-Bcl-2 interactions, to protect against apoptosis, thereby suggesting its value as a new therapeutic target [27,68,69]. Further study is warranted to analyze the function of different ERβ isoforms and their cellular localization, which is essential to completely understand the role of the ERβ in lung cancer. According to the study of Kadota, although nuclear ERα expression was observed in only 17% of the patients with pT1a lung adenocarcinomas, it was an independent predictor of recurrence [61]. The nuclear ERα expression positively correlated with the tumoral FoxP3^+^ lymphocytes, and poor prognostic immune microenvironments.

The G-protein-coupled estrogen receptor (GPER), discovered in 2005, was proposed to be involved in the cancer cell proliferation, migration and invasion, and acts as a modulator of the neoplastic transformation [4,70,71]. It is not only located in the cell membrane, it has also been detected in the Golgi apparatus and endoplasmic reticulum [72,73]. Increased expression of the GPER was observed in the lung cancer cell lines as well as the human and mice lung cancer tissue, and more was located in the cytoplasm [74,75]. Paradoxically, the antagonists/modulators of the classical estrogen receptors such as tamoxifen, raloxifen and fulvestrant, were found to be the GPER agonists [70].

In contrast with GPER, the classical ERs do not contain a hydrophobic part that may serve as a transmembrane domain. However, the presence of ERs in the membrane of somatic and cancer cells have been reported. The membrane translocation of the ERs is mediated by the SRC family of tyrosine kinase [76]. Specific motifs and modifications are required. The knowledge on how the classical ERs translocate to the membrane together with the knowledge on the GPER action, and the interactions between the GPER and the classical ERs is of the greatest importance to understand the membrane-associated non-genomic pathways of estrogen.

**Figure 1:**
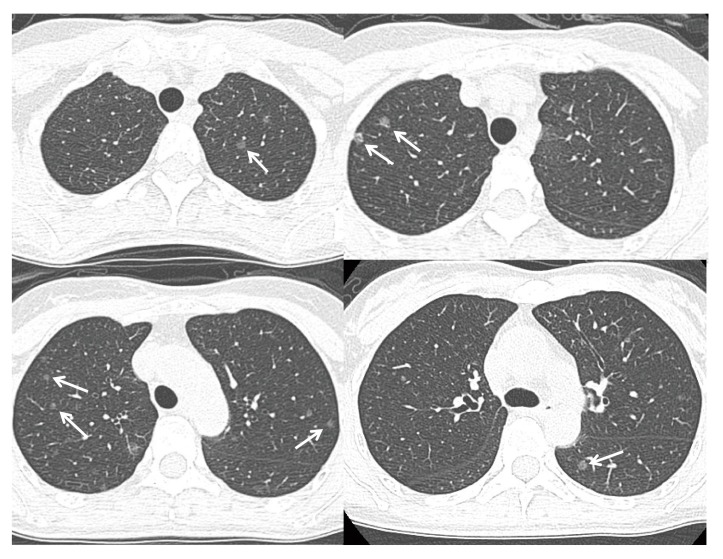
A 48 year-old non-smoking woman was found to have multiple subcentimetre ground glass opacities (arrows) in her bilateral lungs on a low-dose CT screening. Video-assisted thoracoscopic surgery with a right upper lobe wedge resection confirmed the diagnosis of synchronous multiple lung adenocarcinomas harboring the EGFR wild-type.

In premenopausal women, estrogens produced by their ovaries play a major role in the female reproductive organs through the ERα. In postmenopausal women, however, estrogens produced/activated by peripherally localized estrogen-metabolizing enzymes, such as aromatase, which converts androgen into estrogens, are thought to play physiologically and pathologically important roles in various organs through the ERβ, distributing systemically [77]. Estrogen can be synthesized in situ in lung cancer. Ikeda et al. measured the estrogen concentrations in the noncancerous peripheral lung tissue using liquid chromatography/electrospray tandem mass spectrometry in the postmenopausal female patients with synchronous multiple lung adenocarcinomas, and found a significantly higher level than the control cases with a single lung adenocarcinoma [78] (Figure 1). Our study of the malignant pleural effusion of lung adenocarcinoma revealed that some postmenopausal women had extraordinarily high pleural fluid estradiol concentrations, and there was no correlation between the pleural fluid concentrations of estradiol and the vascular endothelial growth factor, a marker of pleural vascular hyperpermeability [79]. In addition, the EGFR wild-type lung adenocarcinoma is probably an estrogen-dependent carcinoma, as a higher expression and potent poor prognosticator of aromatase and the ERβ in the group [62].

Thyroid transcription factor 1 (TTF-1) expression, as a lineage marker of the terminal respiratory unit, is helpful to distinguish the primary (TTF-1 positive) from the metastatic (usually TTF1 negative) lung adenocarcinoma, the pleural lung carcinoma (TTF-1 positive) from the mesothelioma [80]. The TTF-1-positive adenocarcinomas had a statistically significant prevalence of the female, non-smoker, and associated with the EGFR mutation [81,82]. The ER and TTF-1 immunoreactivity is commonly used as a means of distinguishing breast carcinomas from the adenocarcinomas of other primary sites, including the lung, but mostly using the antibody of the ERα. The TTF-1 positivity may be associated with the ERβ expression in lung adenocarcinoma with clinical significance, which therefore deserves further study [83].

## 3. Hormone Replacement Therapy and Lung Cancer Risk and Survival

There were also controversies in the relationship between the hormone replacement therapy (HRT) and lung cancer. Although most studies reported estrogen or HRT adversely affected the prognosis of lung cancer patients [22,23,24,25,26,27,28,29,30], some reported HRT decreased the risk and favorably affected the prognosis [31,32,33,34,35,36]. In the Women’s Health Initiative Trial, HRT using estrogen plus progestin in postmenopausal women did not increase the incidence of lung cancer, but increased the risk (60%) of dying from NSCLC [29]. Unlike the use of estrogen plus progestin, the usage of conjugated equine estrogen alone did not increase the incidence or death from lung cancer [36]. In another Vitamins and Lifestyle Study, postmenopausal women taking estrogen plus progestin were reported to have a 50% increased risk of incident lung cancer for usage of 10 years or longer and an advanced stage at diagnosis [30]. Greiser et al. made a systemic review and meta-analysis from 18 studies for the risk of lung cancer after HRT [84]. Ever use of HRT in non-smoking women may well increase the risk of lung adenocarcinoma. Data from the randomized controlled trials suggested that estrogen/progestin therapy increased the lung cancer mortality. The increased risk of death from lung cancer during the estrogen plus progestin usage in the Women’s Health Initiative Trial was recently reported attenuated after the discontinuation of the medication in a 14-year cumulative follow-up [85].

Siegfried and Stabile provided explanations for the discrepancy of the HRT effect [49]. Different influences of estrogen on the balance of differentiation induction and proliferation in normal lung epithelium and malignant epithelium have been reported [55]. Compared with the matched normal lung tissues, ERβ is overexpressed in lung cancer, which could lead to an abnormal response to estrogen. The ability of the immune system to reject the malignant lung tissues during the early process could be enhanced by HRT [49] and related to a different level of ER expression [61,86,87]. Exogenous hormone usage reduces the local estrogen production by inhibiting the pulmonary aromatase expression. The exact HRT used, i.e., type, duration, timing, and adjusted covariates may modulate the effects of HRT. More specifically designed studies to address the HRT type, smoking, and histology, are therefore warranted to arrive at the more definitive conclusions. However, since the HRT is now recommended to be used for a limited duration, its effects on lung cancer risk or survival may be less pronounced in the future.

## 4. ER as Targets for Lung Cancer Therapy and Relationship with EGFR

Estradiol is locally produced in the NSCLC mainly by aromatase, which is localized in both the epithelial cell components of lung tumors as well as in the infiltrating macrophages; even exclusively confined to the inflammatory cells infiltrated in the pre-neoplastic and neoplastic areas in some of the animal models [88,89]. Patients whose tumors harbored a higher expression of aromatase and ERβ have a lower survival rate, especially in postmenopausal women [13,90]. The use of selective ER modulators and/or aromatase inhibitors have been reported to be clinically effective in the NSCLC that are positive for both the ER and aromatase [91,92]. Recently, Hamilton et al. utilized a quantitative high-throughput screening of approved drugs, and identified the ER antagonist, fulvestrant, as being capable of reducing the mesenchymal features of lung cancer cells and sensitize to the cytotoxic effect of the chemotherapy [93].

**Figure 2:**
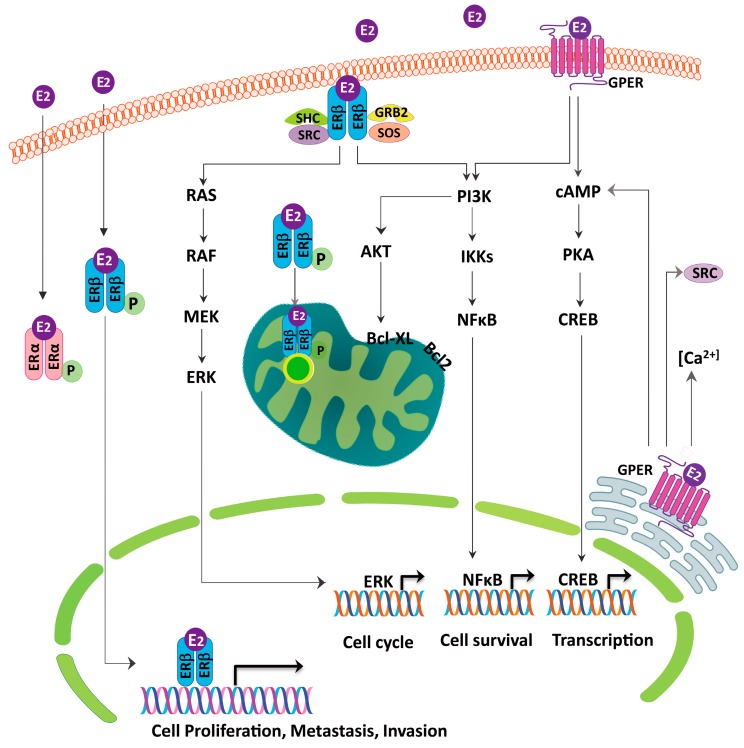
The putative role of the estrogen receptor in regulating the lung cancer cells growth. The estrogen receptor β (ERβ) appears to be the predominant form in lung cancer and is present in the cytoplasm, nucleus, mitochondria and plasma membrane. The ERβ has been found to activate the PI3K/IKK/NFκB, PI3K/AKT/Bcl-XL and the RAS/RAF/MEK/ERK signaling pathways to regulate the cell proliferation, invasion, metastasis, mitochondrial biogenesis and anti-apoptosis. The G-protein-coupled estrogen receptor (GPER) activates the cAMP/PKA/CREB and the PI3K/IKK/NFκB signaling pathways and acts as a modulator of the neoplastic transformation.

**Figure 3:**
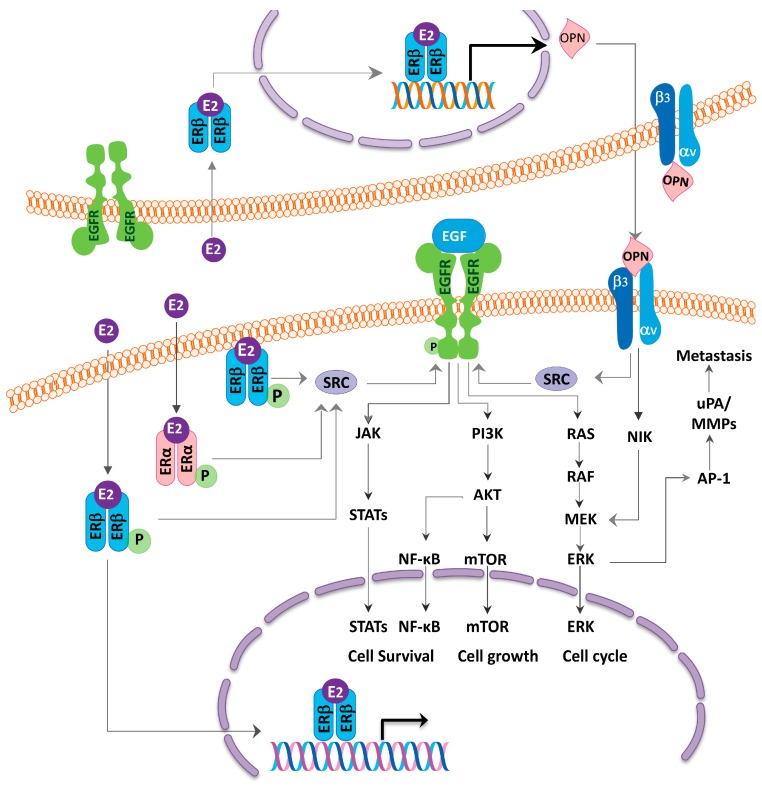
The schematic diagram illustrating the mechanisms of how the estrogen receptor (ER) coordinates with the epidermal growth factor receptor (EGFR) to affect the cell growth in the lung adenocarcinoma. Estrogen stimulates the steroid receptor coactivator (SRC) protein, which in turn, activates the EGFR signaling pathways. In addition, estrogen upregulates the osteopontin (OPN) expression and promotes the lung cancer cell migration via the MEK/ERK signaling pathway. The SRC and OPN contribute to the cross-talk between the ER and the EGFR.

As aforementioned, the activities of the ERβ could be genomic or non-genomic [94] (Figure 2). The estrogen-ERβ complex binds to the nuclear estrogen response elements directly or through the transcription factor, to promote the gene expression. Estrogen also combines with membrane-bound ERβ to activate the cytoplasmic signaling pathway and interacts with the EGFR signaling pathways [95,96] (Figure 3). EGFR has been reported to directly phosphorylate ER at specific serine residues (a ligand-independent signaling) in 87.5% of the ER-positive lung tumors [96,97]. In addition to the MEK/ERK signaling pathway, estrogen also activates the PI3K/AKT signaling pathway, another downstream pathway of the EGFR activation, to promote lung cancer cell metastasis through epithelial mesenchymal transition [98]. Other non-genomic activities have also been explored. Fan et al. found a higher ERβ expression in the lymph node as compared to the primary tumor tissues, and estrogen promotes the lung cancer cell metastasis via the ERβ-mediated up-regulation of the matrix-metalloproteinase-2 [99]. In the mRNA analyses, when comparing the high versus low ERβ expressing tumors by the group of Siegfried and Stabile, the top differentially expressed genes in the high ERβ tumors involved the fibroblast growth factor signaling and the human embryonic stem cell pluripotency [100].

ER and EGFR, as targets for dual lung cancer therapy, have been studied. A combination of the ER antagonist and the EGFR tyrosine kinase inhibitor has been shown to decrease cell proliferation and tumor growth more than one individual treatment in both in vitro and in vivo studies [53,97,101,102]. In the NSCLC cell lines, the EGFR protein expression was down-regulated in response to estrogen and up-regulated in response to anti-estrogens in vitro. Conversely, the ERβ expression is decreased in response to the epidermal growth factor and increased in response to gefitinib [101]. A strong association has been reported between the expression of the ERβ and EGFR mutations in lung adenocarcinoma [53,54,103,104]. These studies have provided evidence of a functional interaction between the ER and EGFR pathways in lung cancer and have supported a rationale to use the combined therapy [95,105].

The available strategies to target the estrogen signaling pathway include the aromatase inhibitors, the reversible nonsteroidal agents (e.g., letrozole, anastrozole) or the irreversible steroidal inactivator (e.g., exemestane), the nonsteroidal elective ER modulator (e.g., tamoxifen, raloxifene), and the ER antagonists (e.g., fulvestrant) [47]. Giovannini et al. reported that the additional effect of letrozole in a patient with lung adenocarcinoma and scalp metastasis persisted on gefitinib [106]. A pilot study revealed that treatment combining the gefitinib and fulvestrant for postmenopausal women with advanced NSCLC was well-tolerated and demonstrated a result [107]. Several phase II clinical trials are currently ongoing to investigate their effects on advanced NSCLC, mostly in a second-line setting and combined with the EGFR tyrosine kinase inhibitor [108] (Table 2). Some studies have included correlative tissue analysis of the ER and the progesterone receptor status to evaluate their role as a predictor of response. Besides the treatment strategies through inhibiting estrogen synthesis or blocking its effect, dexamethasone has been demonstrated to induce the estrogen sulfotransferase to decreases the estradiol levels in tumor tissues and suppress the A549 xenograft tumor growth [109,110].

No significant difference in the clinicopathological characteristic between the ERβ-positive and ERβ-negative lung adenocarcinoma has been mentioned, except some reported that the ER expression correlated with the tumor differentiation [111]. Detailed pathologic examination of the ERβ-positive adenocarcinoma may be necessary to show the genotype-phenotype correlations, similar to those found in the ALK-rearranged or the EGFR-mutated adenocarcinoma [112,113]. Patients with these characteristic histologic features might be good candidates for, and could benefit from, therapy targeting the ER signaling pathways.

Novel technologies, e.g., next generation DNA sequencing, epigenetics, transcriptomics, proteomics, and metabolomics, can make an abundant contribution in the understanding of lung cancer [114,115,116]. The Genetic Epidemiological Study of Lung Adenocarcinoma (GELAC) in Taiwan had found that the gene polymorphisms related to the estrogen biosynthesis and metabolism was associated with an increased occurrence of L858R mutation of the EGFR in non-smoking female lung adenocarcinoma patients [117]. The use of HRT may modify the association of protective EGFR single nucleotide polymorphisms (SNPs) with lung adenocarcinoma risk [118]. The EGFR SNPs have a cumulative effect on decreasing the lung adenocarcinoma risk in non-smoking women with HRT. The ER gene SNPs are associated with a lung adenocarcinoma risk in non-smoking women [119]. The joint effects of the ER and EGFR gene SNPs and HRT usage on lung adenocarcinoma risk highlight the gene-environment interaction in lung carcinogenesis.

## 5. Smoking Aggravates the Effect of Estrogen and Endocrine Disruptive Chemical Targeting ERβ from the Environment May Contribute to the Lung Carcinogenesis

Tobacco smoking is a common source of complex environmental chemical exposure. More than 3000 chemicals have been identified in tobacco smoke, and many of them are both mutagenic and carcinogenic. There exists a phenomena associated estrogenic metabolism with tobacco combustion. Higher levels of polycyclic aromatic hydrocarbon-derived DNA adducts have been reported in female smokers than in male smokers. Estrogen synergize with the tobacco compounds through the induction of CYP1B1, an enzyme responsible for estrogenic metabolism, which leads to enhanced reactive oxygen species formation and carcinogenesis [4,120,121,122].

On the other hand, there have been constant concerns about the endocrine disruptive chemical (EDC) in the environment. EDC that have estrogenic properties are known as xenoestrogens. Although their estrogenic activity is weaker than that of estradiol, newer types of EDC and inadvertent forms of exposure continue to be discovered. There is increasing concern about their cumulative effects in carcinogenesis [123]. Endocrine disruptive chemical, e.g., polychlorinated dibenzo-*p*-dioxins, bisphenol A, polychlorinated biphenyls, polybrominated flame retardants, and methoxychlor, were supposed to be a factor in the environment leading to an increased incidence of lung adenocarcinoma [124,125]. They target ERβ with highly variable effects. Their combination with the aryl hydrocarbon receptor and its nuclear translocator could also modulate the ER activity.

Air pollution containing a mixture of particulate matters (PM) and gas contaminants is generally considered to play a role in the development of lung cancer. According to the particles’ size, they are categorized into coarse particles (<10 and >2.5 μm in aerodynamic diameter, PM10), fine particles (≤2.5 and >0.1 μm in aerodynamic diameter, PM2.5), and ultrafine particles (≤0.1 μm). In addition to the concern of particle size, the combustion of fossil fuels, road traffic, industries, and waste dumps, are known to emit a number of different mutagens and carcinogens, many of which possess xenoestrogenic activity [126,127]. Multifactorial risk assessment incorporating personal exposure history, genetic polymorphisms related to estrogen biosynthesis and metabolism, ER polymorphisms, and biomonitoring data collected from the environment may well identify the population at risk. Collaborations between oncology, system biology, and environmental science will provide an important step to elucidate the etiology of lung cancer and help to make the relevant legislation in the future.

## 6. Conclusions

In addition to the well-known drivers of lung cancer, EGFR (55.7%), KRAS (5.2%), BRAF (2.0%), HER2 (0.7%) mutations, and EML4-ALK translocation (9.8%) [128], a body of epidemiological evidence, preclinical in vitro and in vivo studies, and recent data from the clinical trials, support estrogen as an important factor that contributes to lung carcinogenesis, lung cancer growth, metastasis, and affecting the prognosis. Different pathways of the ER activation and interactions with EGFR were proposed. Estrogen, with its receptor, has the potential to be a prognosticator and a therapeutic target in lung cancer. The ER antagonist may well become a new and effective treatment modality for patients with lung adenocarcinoma and an alternative treatment for patients with acquired resistance to the EGFR antagonists [101,105,129]. However, there were many conflicting results in the literature that need to be addressed [31,32,33,34,35,36,130], of which include the standardized measurements of the ER expression before adopting them as a useful biomarker, the mechanisms that underlie the controversy in the effect of hormone replacement therapy, the role of different estrogen and various ER in lung cancer cell proliferation, migration, and invasion, and the pathways involved in their interactions with other mediators. The risk of EDC exposure also raises the concern of genetic and environmental interaction in lung carcinogenesis.

## Figures and Tables

**Table 1:** Estrogen receptor (ER) detected by immune-histochemical stain as prognosticators in NSCLC.

References	ER Subtype	Location	Prognosis
Kawai 2005 [20]	α	Cytoplasm	Worse
	β	Nucleus	Better
Schwartz 2005 [21]	β	Non-specified	Better (male)
			Worse (female) *
Wu 2005 [51]	β	Nucleus	Better
Skov 2005 [52]	β	Nucleus	Better (male)
			Worse (female)
Nose 2009 [53]	α	Cytoplasm	Worse
	β	Nucleus	Better
Raso 2009 [54]	β	Nucleus	Worse
Stabile 2011 [55]	β	Cytoplasm	Worse
Rouquette 2012 [56]	α	Nucleus	Better
Rades 2012 [57]	α	Non-specified	Worse
Karlsson 2012 [58]	β	Nucleus	Better
Navaratnam 2012 [59]	β1	Nucleus	Better in earlier stage
			Worse in later stage
Liu 2013 [60]	β2,5	Cytoplasm	Better
Kadota 2015 [61]	α	Nucleus	Worse
Liu 2015 [62]	β	Cytoplasm	Better
Skjefstad 2016 [63]	β	Nucleus	Worse (female)
Tanaka 2016 [64]	β	Non-specified	Worse (male)

* Not significant but with a trend.

**Table 2:** Clinical trials of hormone therapy in advanced NSCLC (http://www.clinicaltrial.gov/, accessed on 11 June 2017).

Patient Population	Allowed Prior Therapy	Treatment	Correlate Response with Receptors Expression	ClinicalTrials.Gov Identifier & Status
Stage IIIB or IV NSCLC, both gender	≥1 prior chemotherapy	Erlotinib + fulvestrant vs. Erlotinib	Yes	NCT00100854 Active, not recruiting (2004~)
Stage IIIB or IV NSCLC, both gender, ER or PR positive	Stable disease on erlotinib >2 months, prior chemotherapy not defined	Erlotinib + fulvestrant (single arm)	Before trial entry	NCT00592007 Terminated with results (2007~)
Stage IIIB or IV, postmenopausal women	Completed 4 cycles of induction platinum-based chemotherapy	Arm B-1. Best supportive care (BSC); Arm B-2. BSC + bevacizumab; Arm A-1. Fulvestrant + anastrozole; Arm A-2. Fulvestrant + anastrazole + bevacizumab	Yes	NCT00932152 Terminated with results (2010~)
Stage III or IV NSCLC, postmenopausal women	Chemotherapy, 0–1 line for EGFR mutations and 1–2 lines for EGFR wild type	Gefitinib + fulvestrant vs. Gefitinib for EGFR mutations; Erlotinib + fulvestrant vs. Erlotinib for EGFR wild type	No	NCT01556191 Recruiting (2012~)
Stage IV NSCLC, postmenopausal women	Phase I dose escalating study	Exemestane + premetrexed, carboplatin	No	NCT01664754 Active, not recruiting (2012~)
Stage III or IV NSCLC, postmenopausal women	Chemotherapy 1–3 line	Exemestane (single arm)	No	NCT02666105 Recruiting (2016~)

* In the order of study start date.

## Abbreviations

EDC	Endocrine disruptive chemical
EGFR	Epidermal growth factor receptor
ER	Estrogen receptor
ERα	Estrogen receptor α
ERβ	Estrogen receptor β
GPER	G-protein-coupled estrogen receptor
HRT	Hormone replacement therapy
NSCLC	Non-small cell lung cancer
OPN	Osteopontin
PM	Particulate matters
SCLC	Small cell lung cancer
SNPs	Single nucleotide polymorphisms
